# Satellite mapping of maize cropland in one-season planting areas of China

**DOI:** 10.1038/s41597-023-02334-5

**Published:** 2023-07-07

**Authors:** Qi Xin, Liqiang Zhang, Ying Qu, Hao Geng, Xingang Li, Shuwen Peng

**Affiliations:** Faculty of Geographical Science, Beijing Normal University, Beijing, China

**Keywords:** Agriculture, Geography

## Abstract

As the major maize-cultivated areas, the one-season cropland of China is increasingly threatened by rapid urbanization and soybean rejuvenation. Quantifying the area changes of maize cropland is crucial for both food and energy security. Nonetheless, due to the lack of survey data related to planting types, long-term and fine-grained maize cropland maps in China dominated by small-scale farmlands are still unavailable. In this paper, we collect 75,657 samples based on field surveys and propose a deep learning-based method according to the phenology information of maize. With the generalization capability, the proposed method produces maize cropland maps with a resolution of 30 m from 2013 to 2021 in the one-season planting areas of China. The maize-cultivated areas derived from the maps are highly consistent with the data recorded by statistical yearbooks (*R*^2^ = 0.85 on average), which indicates that the produced maps are reliable to facilitate the research on food and energy security.

## Background & Summary

As one of the most widely distributed staple food, maize is grown in at least 164 countries and produces more than 35 percent of the world’s food^1,2^. Due to its rich starch content and comparatively easy conversion to ethanol, maize is not only important for global food security but also a popular feedstock for biofuel production all over the world. According to the statistical corporate database released by the food and agriculture organization, China has become the second biggest maize-producing country, contributing around 23% of the production of global maize^3^, which is crucial for both food and energy security. Nonetheless, due to rapid urbanization and industrialization, global temperature rises, soybean rejuvenation, as well as changing precipitation patterns, the one-season cropland of China, which produce maize with high nutritional value, are at risk^4,5^. Therefore, mapping the one-season maize cropland in China will be of great significance to ensure food and energy security^6,7^.

Over the last decades, several methods have been proposed to map maize with optical remote sensing^8–12^. Traditionally, most maize maps are generated by performing crop classifications with MODIS satellites in the 500 m to 1 km coarse spatial resolution range. Although daily MODIS data can provide continuous phenological information of crops with a high temporal resolution, its coarse spatial resolution cannot accurately capture the crop distributions in China dominated by small-scale farmlands. To address the challenge, a plausible solution is to generate maize maps with high-spatial resolution satellites such as Sentinel and Landsat. Although the spatial resolution of Landsat images is lower than that of the Sentinel images, Landsat can provide long-term earth observation over 50 years, which has become the indispensable foundation for long-term and large-scale land use analysis.

A notable example of maize mapping studies using Landsat is the CropScape, displaying data from the United States Department of Agriculture (USDA) Cropland Data Layer (CDL), which adopted machine learning methods to generate maps with more than a hundred crops including maize, across United States^13^. Yang *et al*.^14^ generated the distributions of maize in Heilongjiang province from 2015 to 2016 by applying a random forest model on Landsat 8 images. However, the model cannot be readily extended to produce maps in other years due to its limited generalization ability. Based on the time-weighted dynamic time-warping method, the distributions of maize in China from 2016 to 2020 were estimated with both Landsat and Sentinel images^15^. Nonetheless, since the Sentinel satellite was launched by ESA in 2015, the method cannot be used to generate maps before 2016. Moreover, the accuracy of the product was limited. To our knowledge, due to the lack of training samples and the variations of plants, there is no long-term maize mapping product in China with 30 m or higher resolution, which may hinder the management of food and energy security for the second-largest maize producer.

To address the challenges, we propose a data synthesis and deep learning-based method to produce long-term and large-scale maize mapping in Northern part of China with Landsat images. Our contributions are three-fold. First, we collected 75657 samples with ground truth labels based on field surveys conducted from 2017 to 2021. Second, a data synthesis method is proposed based on the crop phenology of maize in the study areas to alleviate the spatial and temporal variations of plants. Third, an LSTM-based deep learning method is proposed to produce maize maps with high accuracy. The proposed method with generalization capability is applied to Landsat images to produce maize maps from 2013 to 2021 in the one-season cropland of China. The areas derived by the maize maps are highly consistent with the data recorded by statistical yearbooks^16^ (*R*^2^ = 0.85), which indicates that the proposed method is able to generate maize maps with high accuracy in China dominated by small-scale farmland.

## Methods

### Study area

**Fig. 1:**
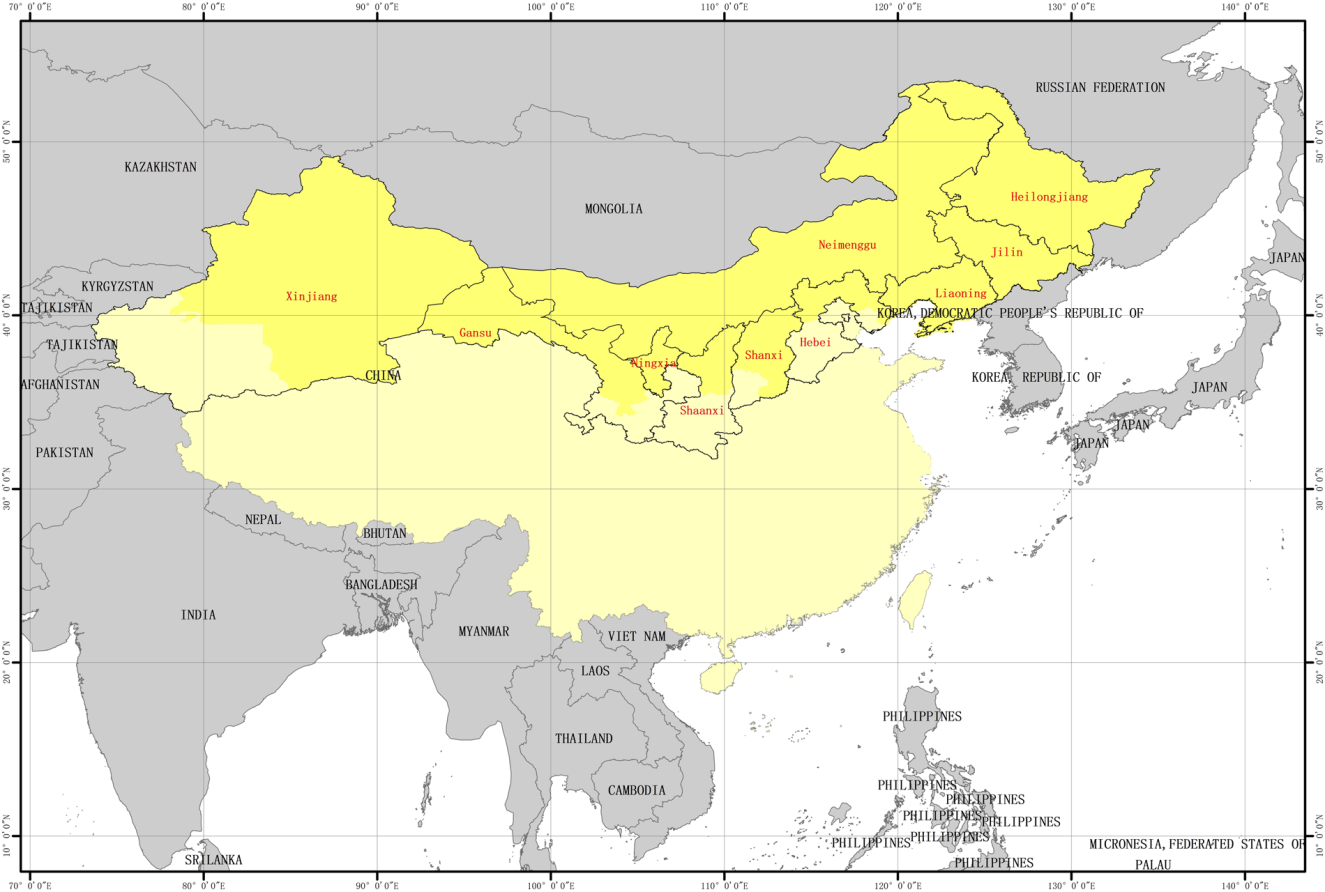
Geographic distributions of the maize maturity in the one-season maize cropland of China.

Our study areas are located in the one-season maize cropland of China, which tends to produce maize with high nutritional value, and is convenient for crop management. As shown in Fig. 1, the crop areas are divided according to the Chinese crop maturity distribution products released by Miao Zhang *et al*. in 2017^17^ and China’s administrative divisions^18^. The study includes maize cropland in 10 provinces, i.e., Heilongjiang, Jilin, Liaoning, Neimenggu, Hebei, Shanxi, Shaanxi, Ningxia, Gansu, and Xinjiang. Based on the study of crop phenology in China^19^, the phenological periods of maize in the study areas are close, i.e., the phenological time differences of the growth stages among maize in the study areas are within one month.

### Overview of the maize classification method

**Fig. 2:**
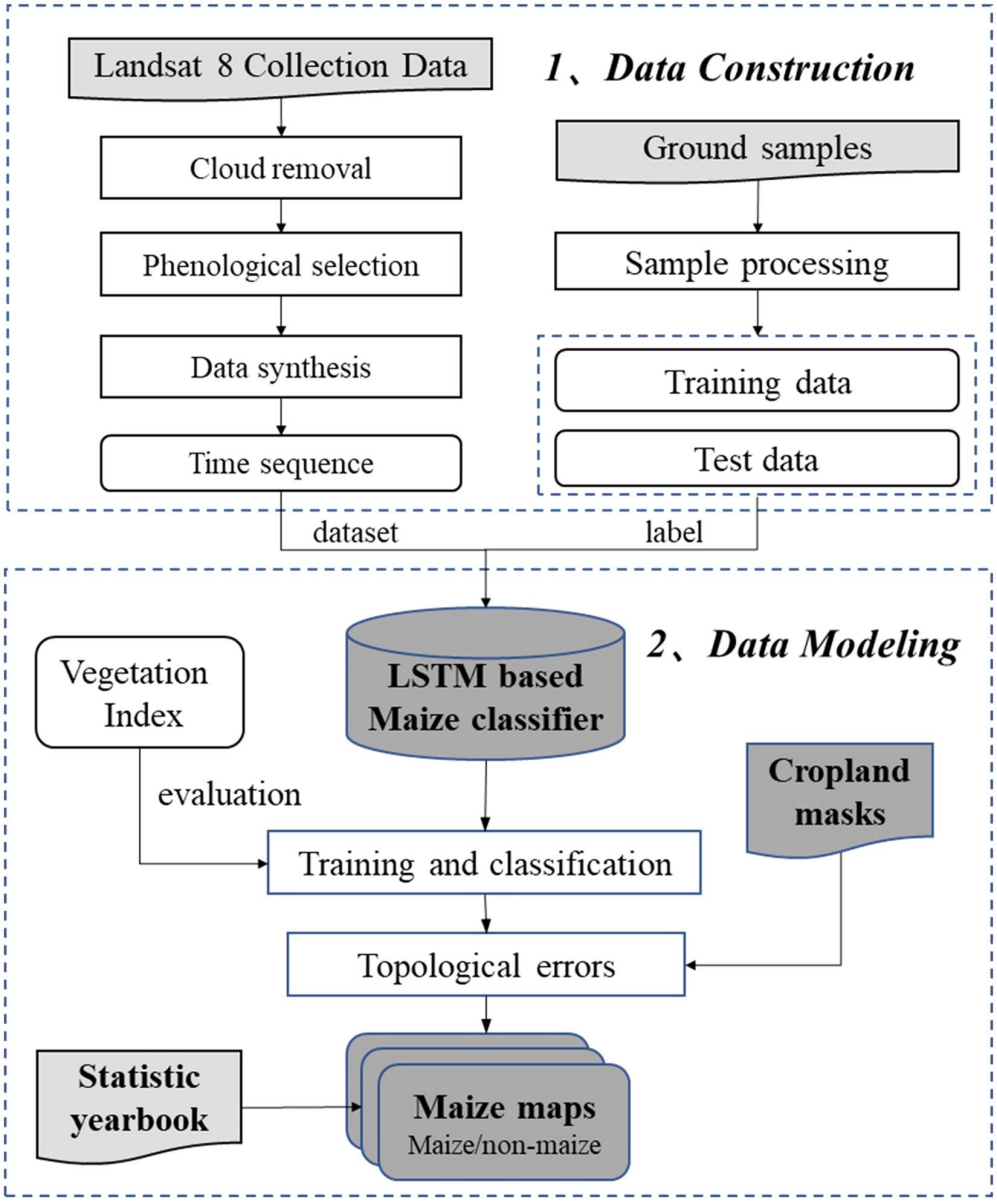
The maize cropland classification workflow.

Figure 2 shows the workflow of the proposed method consisting of data construction (image pre-processing and sample collection), and data modeling (deep-learning-based maize classification and noise removal). (1) The image pre-processing step includes cloud removal, phenological selection, and image synthesis steps. Images representing crop growing and harvesting periods are collected and synthesized based on the phenology calendar of maize to alleviate the spectral variability of crops. More details are provided in the ‘Landsat-8 images and pre-processing’ section. (2) In the sample collection step, we collected 75,657 samples from synthetic images with ground truth labels based on field surveys conducted from 2017 to 2021. More details are described in the ‘Sample collection’ section. (3) In the classification step, we proposed an LSTM-based classification model. The model is optimized with training samples and applied to Landsat 8 images to map long-term maize landcover for different years. (4) In the noise removal step, to further improve the classification accuracy, we use cropland masks to exclude misclassified non-crop pixels and remove speckle noise in crop maps. More details can be found in the ‘Noise removal’ section.

### Landsat-8 images and pre-processing

We use the surface-reflectance (SR) images^20^ acquired during 2013–2021 with the Landsat 8 multispectral instrument as the input data for crop classification tasks, since SR is able to alleviate the spectral variability of crops, which is reliable for cross-domain image classification tasks, such as rice mapping, yield mapping, sugarcane identification and planting intensity monitoring^21–23^.

### Cloud removal

Due to the limitations of spatial resolution, it is hard to identify crops with features extracted from a single Landsat image. Thus, the input data is synthesized by collecting a sequence of images according to the phenology of maize in the study areas. Before data synthesis, the cloudy images are removed based on the cloud cover provided by Google Earth Engine (GEE). After cloud removal, we found that 5% of the images were still covered by clouds. To further improve the accuracy of the classification model, we replaced the cloud-contaminated image bands with clean image bands captured at a similar time, which increases the differences of the spectral features between maize and other plants, especially non-crops.

### Phenological selection and image synthesis

**Fig. 3:**
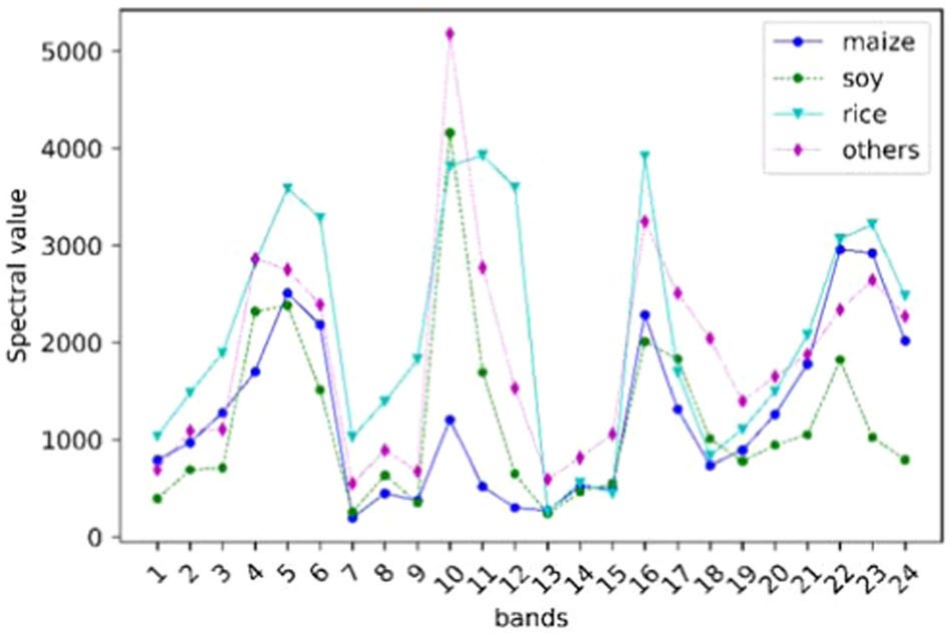
The reflectance of crops in different spectral bands of synthetic images. The locations of the collected samples: maize: 41.010865°N, 124.815502°E (2019); soy: 48.152758 N, 122.393964°E (2019); rice: 122.437692°E, 39.483458°N (2019); others: 125.552640°E, 49.589498°N (2019).

According to the phenology information of maize in one-season cropland of China (Fig. 3), we take images of crops for the growing period and images after crop harvesting as input for maize identification. To improve the generalization capacity of the proposed method, we select images captured from April to November and synthesize a scene every two months for maize classification according to the phenology of maize in the study areas. Each pixel in the synthetic image is created by selecting the maximum value of the pixels covering the same area within two months. In this way, it is easy to extract discriminative and representative features, which are beneficial for classification tasks. The Landsat image has six bands with a resolution of 30 m, including the blue, green, red, near-infrared, shortwave infrared 1, and shortwave infrared 2 bands. After image synthesis, the six-band images of the four periods (from April to November) are superimposed into a 24-dimensional image sequence. The input of the data is a set of multi-variable time series per pixel. Each multi-variable pixel includes the six bands of Landsat from four periods, i.e., the dimension of input is 24.

### Sample collecting

**Fig. 4:**
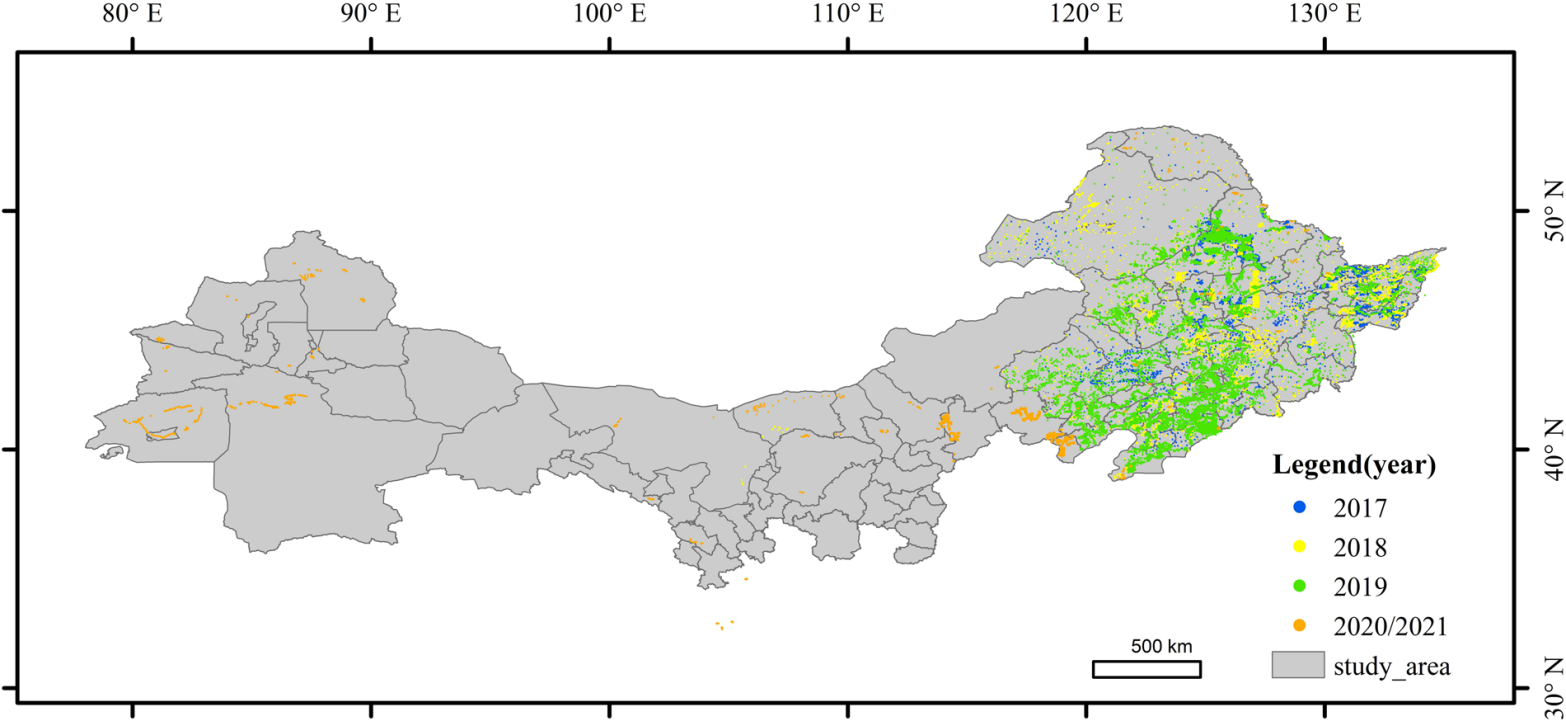
The distributions of the collected samples. To improve the generalization ability of the model with imbalanced training samples, we use to synthesize the images with phenological information to improve the robustness of the model.

We collected samples based on field surveys conducted from 2017 to 2021, as shown in Fig. 4. RGB composites (R: SWIR1, G: NIR, B: Red) were created after the field survey using high-resolution images in Google Earth and Sentinel images. To eliminate misidentified crops and samples located at road or field boundaries, the composite images from April and June are used to exclude errors. In fragmented areas, we vectorized the input sample points with Google high-definition images into a planar land parcel such that it can increase the ability to extract and identify small land parcels. In addition, non-farmland samples were also collected based on the high-resolution images from Google Earth. Finally, for 2017, 2018, 2019, 2020, and 2021, we obtained 16,187, 21,431, 22,171, 8029, and 7839 independent maize and non-maize samples, respectively. All the samples are mixed and randomly divided into training and testing sets with 70% and 30% of the labeled samples, respectively. To enhance the generalization ability of the proposed model, the training samples collected from different provinces are fed into the model to extract the features of different crops with spectral and temporal variations.

### The LSTM-based deep learning classification model

**Fig. 5:**
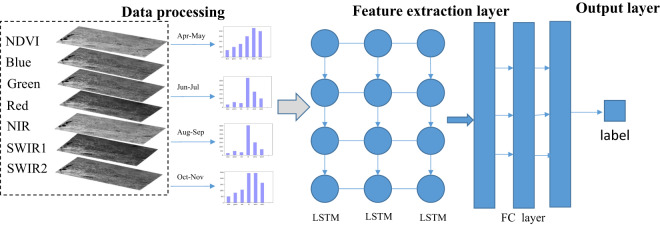
Deep learning model of the maize mapping framework.

The framework of the deep learning model is shown in Fig. 5. The structure includes two modules: the feature extraction module and the classification module. The model aims to categorize each pixel of the input image into one of the given classes^24,25^. For each pixel, the input vector of the model is a 24-dimensional vector.

Besides image bands, traditional methods usually adopted some commonly used vegetation indices to improve the accuracy of the classification model. Thus, we conduct experiments by stacking the NDVI, EVI, and NDWI (defined in Table 1) with the 24-dimensional image sequence as the input data. The results show that the accuracy of the model was not improved. Therefore, in our final model, only the 24-dimensional synthetic images are fed into the network to perform classification tasks. Specifically, it includes 6 bands corresponding to the maximum of 4 periods, namely Blue, Green, Red, NIR, SWIR1, SWIR2. The input of the model is summarized in Table 2.

**Table 1:** Formulation of the three spectral indices evaluated in the study.

Indices	Formulation	Reference
NDVI	${\rm{N}}{\rm{D}}{\rm{V}}{\rm{I}}=\frac{{\rho }_{NIR}-{\rho }_{red}}{{\rho }_{NIR}+{\rho }_{red}}$	^30^
EVI	${\rm{E}}{\rm{V}}{\rm{I}}=2.5\times \frac{{\rho }_{NIR}-{\rho }_{red}}{{\rho }_{NIR}+6\times {\rho }_{red}-7.5\times {\rho }_{blue}+1}$	^30^
NDWI	${\rm{N}}{\rm{D}}{\rm{W}}{\rm{I}}=\frac{{\rho }_{green}-{\rho }_{NIR}}{{\rho }_{green}+{\rho }_{NIR}}$	^31^

**Table 2:** Summary of data characteristics used in the maize mapping model.

Input data	Length of time series data	Number and type of labels	dataset size
6-band (Blue, Green, Red, NIR, SWIR1, SWIR2) Landsat images	4 periods per year	four classes (maize: 23,676, soybeans:15552, rice:8105, others:28324)	Used 33315 Landsat images: 3.98TB Used labels: 75,657

To learn the temporal and spectral dependence information from maize growth stages, we use a 3-layer LSTM to extract spectral-temporal features from image sequences. Each LSTM hidden layer has 128 dimensions. In the classifier model, we use a three-layer fully connected network to convert features into classification probability values. The numbers of nodes are 128, 128 and 64 in three layers, respectively. The first two layers use tanh as the activation function, while the last layer uses a sigmoid activation function to obtain the classification category of the given pixel.

The model uses a cross-entropy function^26^ *L*_*ce*_ as the loss function of the model. In addition, a centre loss *L*_*C*_ is used to maximize the interclass distance of features, and minimize the intraclass distance, which improves the accuracy of maize classification.

1 $${L}_{ce}=-\frac{1}{N}{\sum }_{i=1}^{N}{\sum }_{j=1}^{C}\left({p}_{(j)}logq(j)\right)$$

2 $${L}_{c}=\frac{1}{2}{\sum }_{i=1}^{N}{{\sum }_{j=1}^{C}\parallel \,{f}_{j}-{c}_{j}\parallel }_{2}^{2}$$

3 $$L={L}_{ce}+w{L}_{c}$$

The loss function used in this study consists of two components: the cross-entropy loss function *L*_*ce*_, and the center loss function *L*_*C*_. In *L*_*ce*_, *p*(*j*) is the probability of sample i belonging to class j, *q*(*j*) is the one-hot label of the sample, and N and C are the number of samples and the number of classes, respectively. ${\parallel \,{f}_{j}-{c}_{j}\parallel }_{2}^{2}$ represents the Euclidean distance between i and j. The final loss *L* used in our study is the sum of *L*_*ce*_ and a weighted *L*_*C*_, with the weight w being set to −0.001.

To ensure the sample balance and better feature extraction of different classes, we train a 4-class model and take the other three classes besides maize as non-maize during the evaluation and maize mapping.

As discussed in the sample collection section, all the samples are mixed and randomly divided into training and testing sets with 70%, 30% of the labelled samples, respectively. We choose the adam optimizer to update the network weights, and the initial learning rate is set as 0.001, The batch size for training is set to 20. During the training process, after 200 iterations, the model usually converges and the loss decreases slowly, so we step the training after 250 iterations.

### Noise removal

The proposed classification model is optimized with training samples and applied to Landsat 8 images to map long-term maize landcover for different years. To further improve the maize cropland classification accuracy, after deploying the model on Landsat images, we use the cropland mask to exclude the misclassified non-cropland areas from the maize cropland maps. That is, we utilize the LSTM-based approach to generate maize cropland maps, which may include a small portion of non-croplands being misclassified into maize croplands. To remove the non-croplands, we use the cropland data in the widely used land use and land cover data products as the mask. Next, we overlay the masks with our classification results in the same years, and exclude the non-cropland areas from our classification results. Specifically, we use the cropland cover data from the Globeland30^27^ product in 202030 as the cropland mask to generate the maize croplands in 2020–2021. The cropland cover data in 2015 and 2019 generated by Potapov *et al*.^28^ are used as the cropland masks to generate maize croplands in 2013–2015 and 2016–2019, respectively.

## Data Records

Nine maize cropland with a nominal 30-m resolution are generated for one-season planting areas of China from 2013–2021. These datasets are shared in Geotiff format at figshare^29^. The maize maps contain values 1 and 0, representing maize, and non-maize (including other crops and nonarable land), respectively. The data range from 78.025° to 135.089° longitude and 34.126° to 53.562° latitude. Data maps can be opened in ArcGIS, QGIS, and other similar software.

## Technical Validation

The generated maize maps are evaluated from two aspects, i.e., the accuracy of the proposed method and the consistency between the maize-cultivated areas derived by the proposed method and the ones recorded by the statistical yearbooks^18^. Four matrices, i.e., the (1) Overall accuracy (OA), user accuracy (UA), producer accuracy (PA), and the F1-score (F1) were calculated for the annual maize maps based on the testing samples. (2) The derived areas based on the annual maize maps are compared to the prefecture-level statistical yearbook records from 2013 to 2020. Note that since the 2021 statistical yearbook is not complete, no comparison has been made in 2021.

**Fig. 6:**
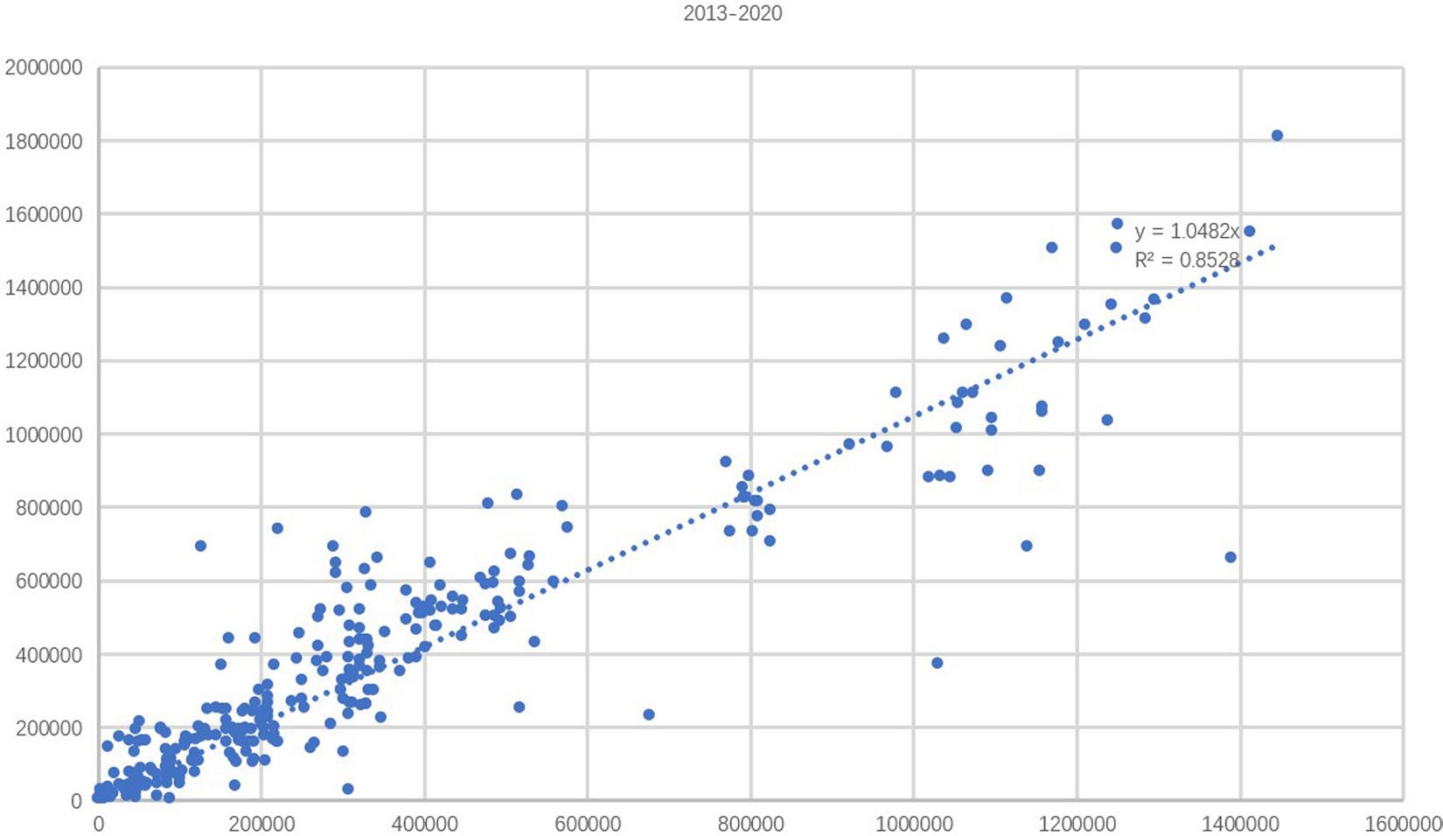
The comparison of the estimated planting areas of maize from our annual crop maps to statistical data at the municipal level for 2013–2020.

We estimated the F-score for maize and non-maize classes to measure the classification performance with precision (UA), recall (PA), F1, and OA, respectively. As shown in Table 3, we can observe that the proposed maps can generate maps with high accuracy across different years. For example, the overall accuracy for all years reaches 89%. To further evaluate the accuracy of the produced maize maps, we compare the areas estimated from maize maps to the ones from the statistical yearbook for 2013–2020, at the prefectural level. As shown in Fig. 6, we can obtain an average *R*^2^ of 0.85, which indicates that the derived maize areas were consistent with the statistical data for all the years.

**Table 3:** The evaluation of maize maps based on ground truth data.

Year	Class	Maize	Non-maize	UA	PA	F1	OA
2017	Maize	531	95	0.88	0.84	0.86	0.87
	Nonmaize	68	1202	0.92	0.94	0.93	
2018	Maize	605	134	0.82	0.82	0.82	0.87
	Nonmaize	130	2345	0.95	0.95	0.95	
2019	Maize	707	127	0.85	0.85	0.85	0.89
	Nonmaize	120	2372	0.949	0.95	0.95	
2020 and 2021	Maize	744	76	0.89	0.907	0.90	0.89
	Nonmaize	89	1473	0.891	0.902	0.89	
2017–2021	Maize	6170	1017	0.87	0.86	0.87	0.89
	Nonmaize	933	10370	0.91	0.92	0.91	

The first four columns show the confusion matrix of the classification results, which indicates the number of times instances of class A are classified as class B. For example, to know the number of times the classifier confused maize with nonmaize in 2017, we would look at the first row and fourth column of the confusion matrix. The last four columns show the UA, PA, F1, and OA of the maize maps, respectively.

As shown in Fig. 6, the total *R*^2^ value from 2013 to 2020 is 0.85. The evaluation *R*^2^ for individual years from 2013 to 2020 are shown in Supplementary Fig. S1. A confusion matrix of the maize maps was also calculated using the collected testing data (Section: training and validation data) to investigate the classification accuracy of the results. We observe that some uncertainty sources may affect the mapping results, mainly due to spectral variations and mixed pixels. The landscape of the study areas is complex and heterogeneous, which may cause the misclassification of maize classes. Although we used the two-month synthesis method to recover the images covered by cloud, the crop identification effect varies due to the difference in phenological dates between two adjacent scenes. In addition, some maize may be mixed with other crops, roads or weeds, since each pixel covers an area of 30 × 30 m, resulting in certain errors in maize identification.

## Usage Notes

**Fig. 7:**
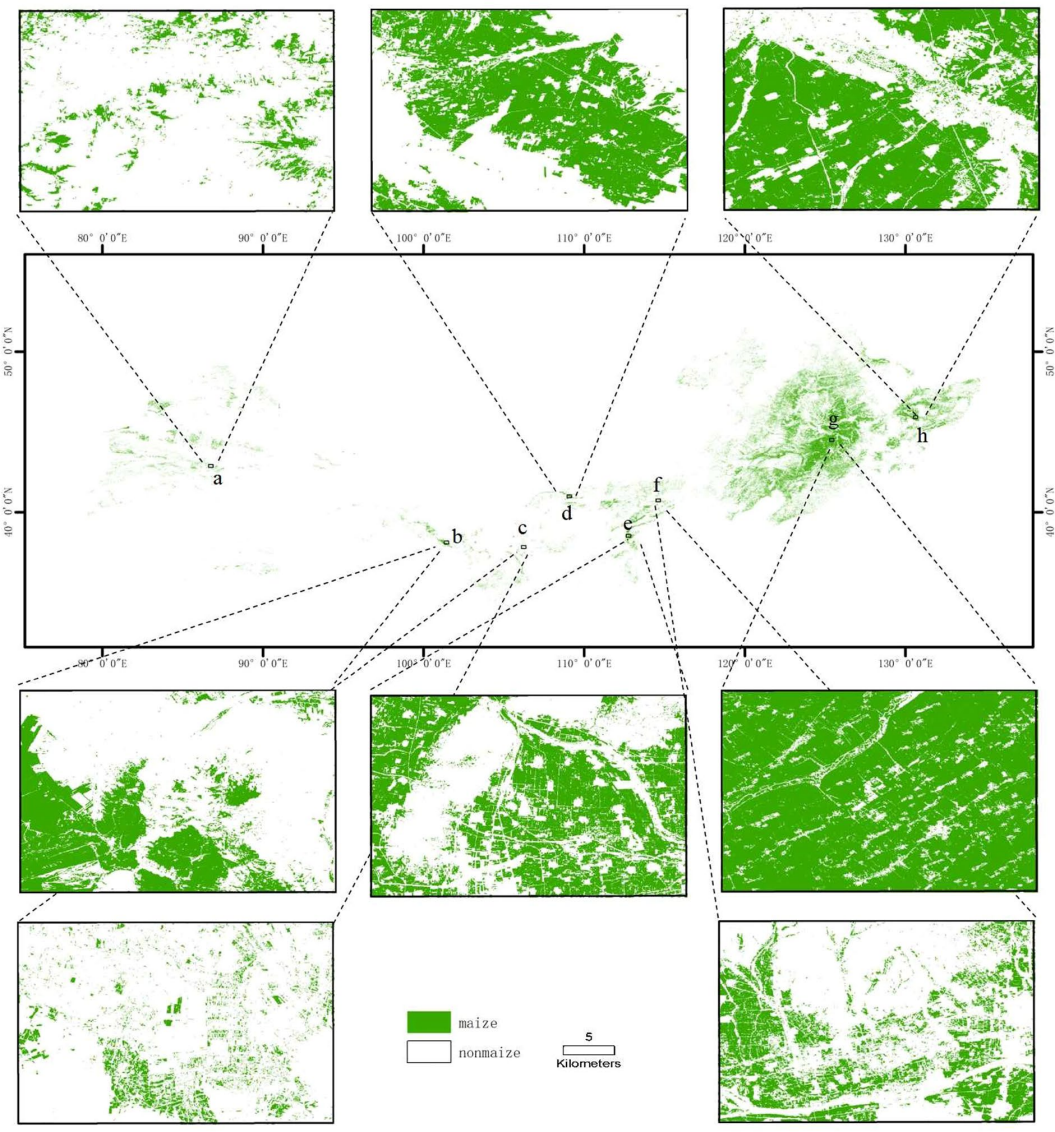
The spatial details of the crop map in 2019 in the one-season cropland of China, include Northwest China and Northeast China. Site a (86.7° E, 46.9° N), b (101.4° E,38.1° N), c(106.2° E,37.8° N), d(109.06° E,41.0° N), e(112.75° E, 38.55° N), f(114.6° E,40.75° N),g(125.4° E,44.5° N) and h (130.6° E, 46.9° N).

As the second biggest maize-producing country in the world, China produced around 23% of the world’s maize production. Nowadays, the cultivated areas of maize in the one-season cropland of China are increasingly threatened by global warming, rapid urbanization and industrialization. Quantifying the changes in the maize planting areas in the Northern part of China is crucial for both food and energy security. In this study, we proposed a simple yet effective maize classification method based on data synthesis and deep learning. With the proposed model, we map long-term maize landcover with a resolution of 30 m from 2013 to 2021 in the Northern part of China (Supplementary Fig. S2). Since the maize-cultivated areas derived from the produced maps are highly consistent with the data recorded by the statistical yearbook, the produced maize maps can be used to support large-scale maize yield estimation. It is worth noting that the proposed method with generalization capacity can also be applied to Landsat images before 2012, the generated annual maps can also track the long-term changes in maize-cultivated areas, which can facilitate the research on water use, soil fertility, and land improvement or degradation in Northeast China, Inner Mongolia, and Xinjiang, as shown in Fig. 7.

## Supplementary information

Supplementary

## Data Availability

Code for downloading Landsat images, processing samples, training models, and producing maps will be available from the Figshare repository^29^.
