# Supplementary material for: Satellite mapping of maize cropland in one-season planting areas of China
Source: Sci Data. 2023 Jul 7;10:437. doi: 10.1038/s41597-023-02334-5 (PMC10328911; doi:10.1038/s41597-023-02334-5)
Supplement: Supplementary file 1 — Supplementary [file 41597_2023_2334_MOESM1_ESM.docx]

Supplementary information

| 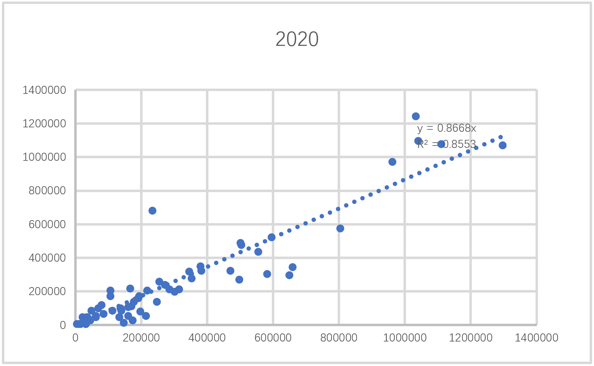 |
| --- |
| 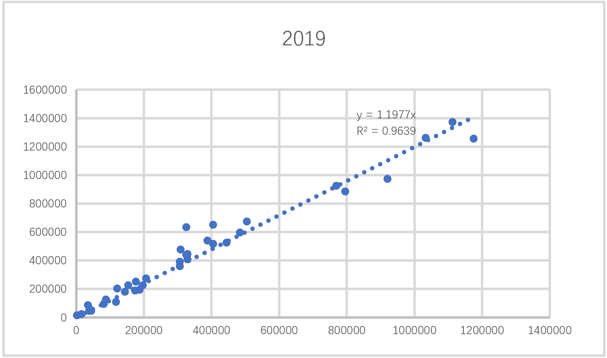  (b) |
| 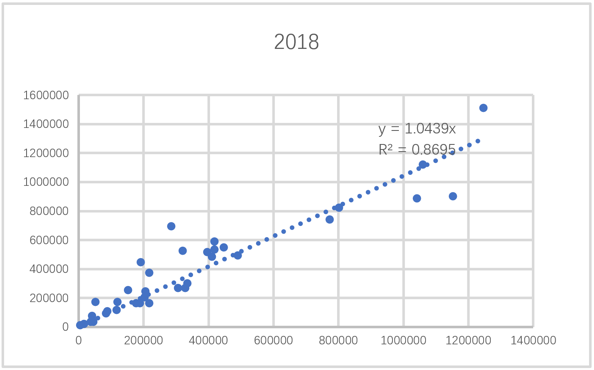  (c) |
| 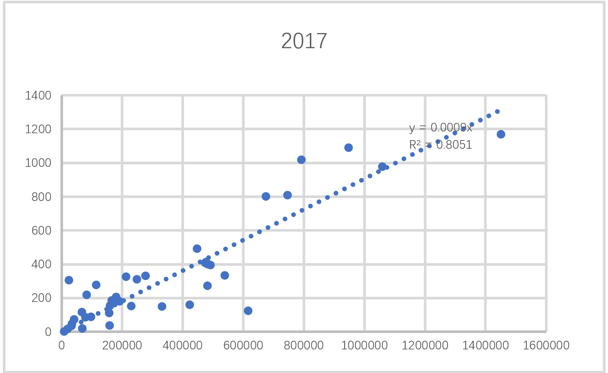  (d) |
| 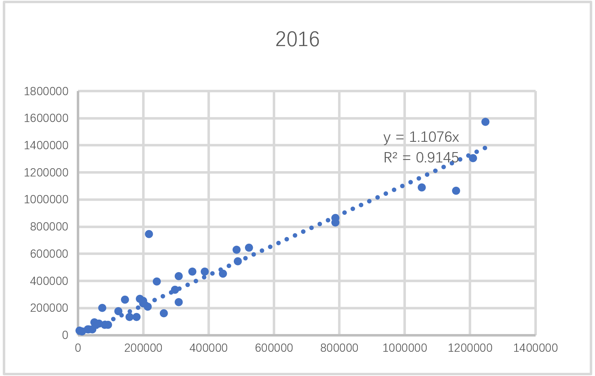  (e) |
| 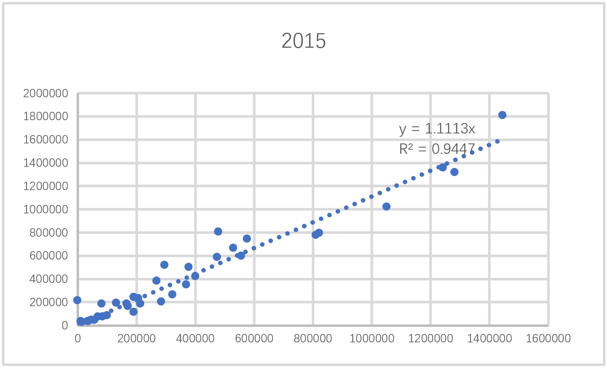  (f) |
| 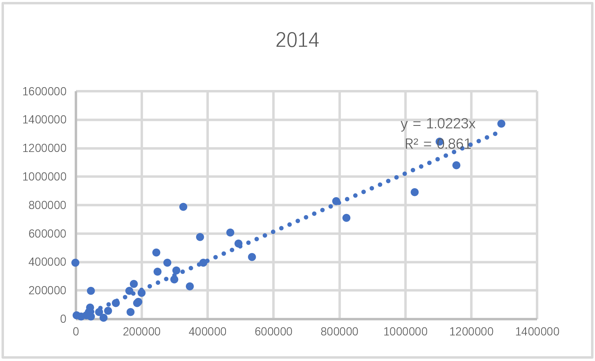  (g) |
| 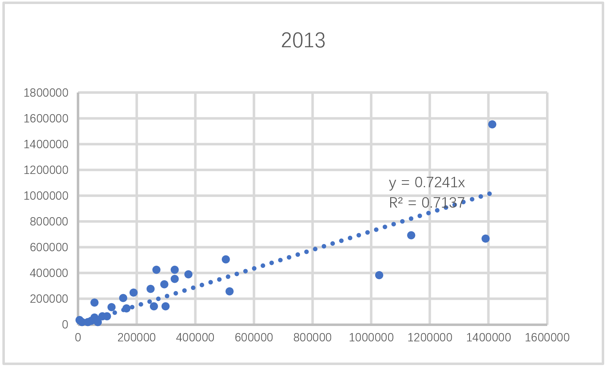  (h) |

**Fig. S1** The comparison of the estimated planting areas of maize from our annual crop maps to statistical data at the municipal level from 2013-2020. (a)-(h) The estimated $R^{2}$ for each year during 2020-2013.


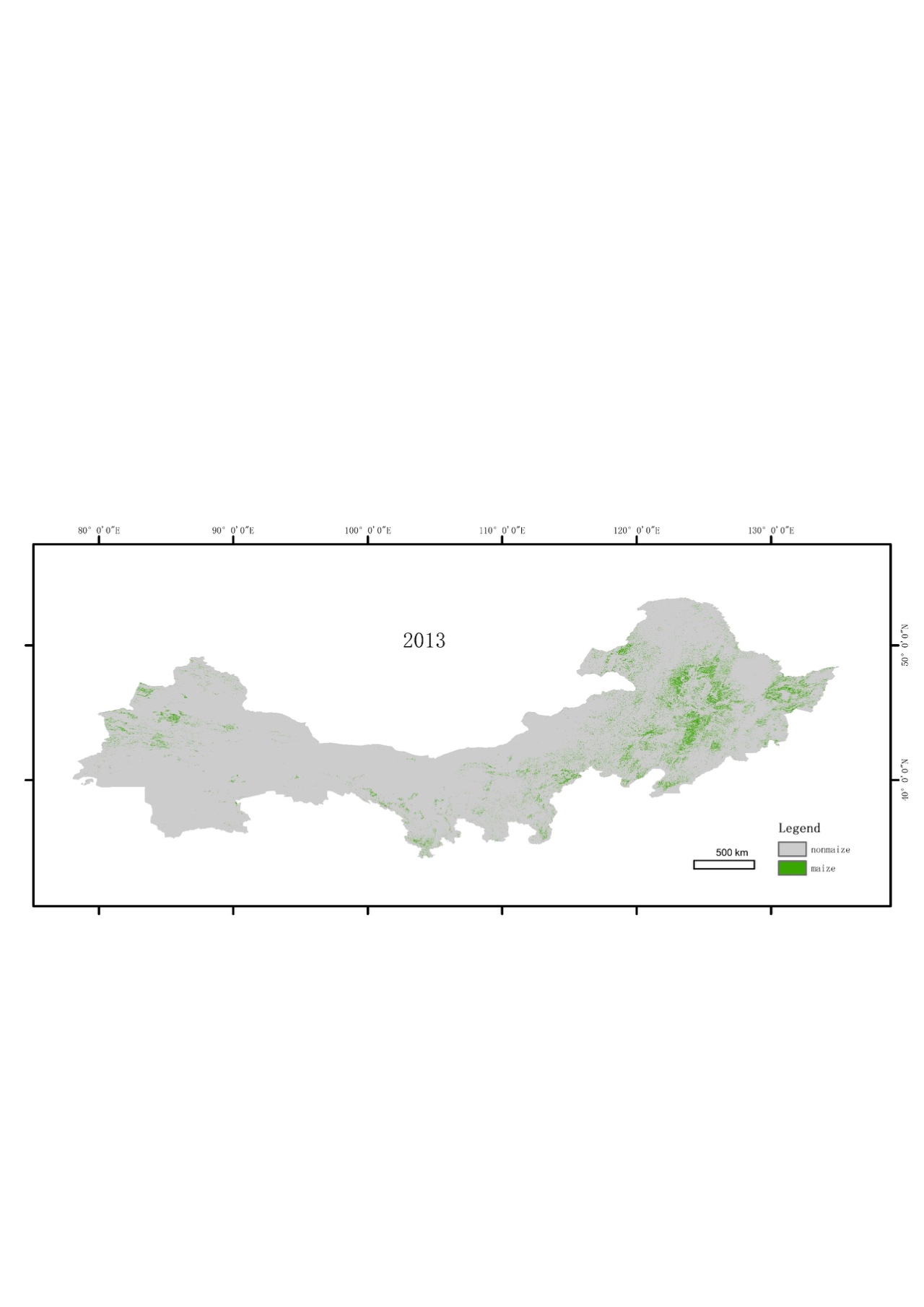


(a)


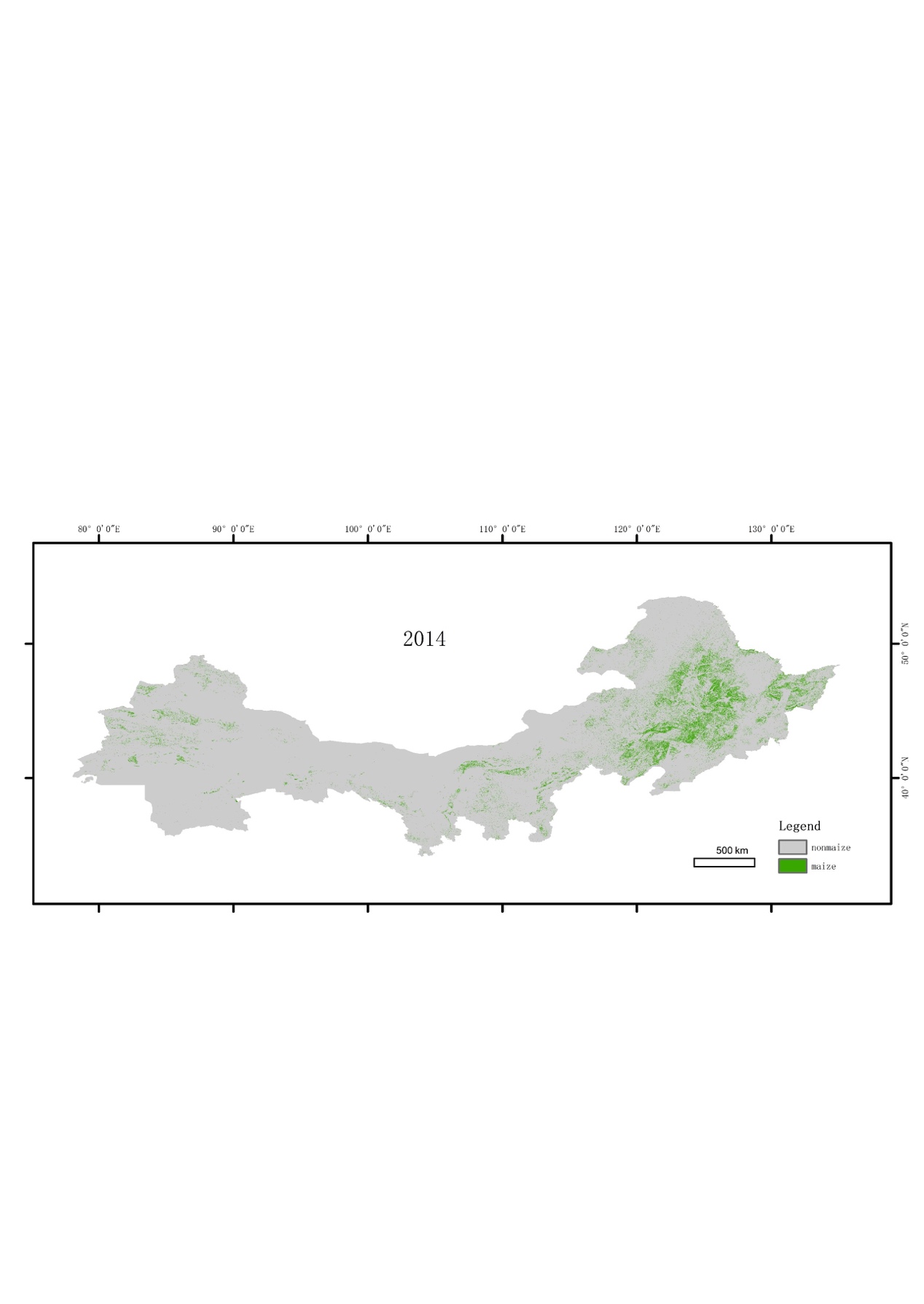


(b)


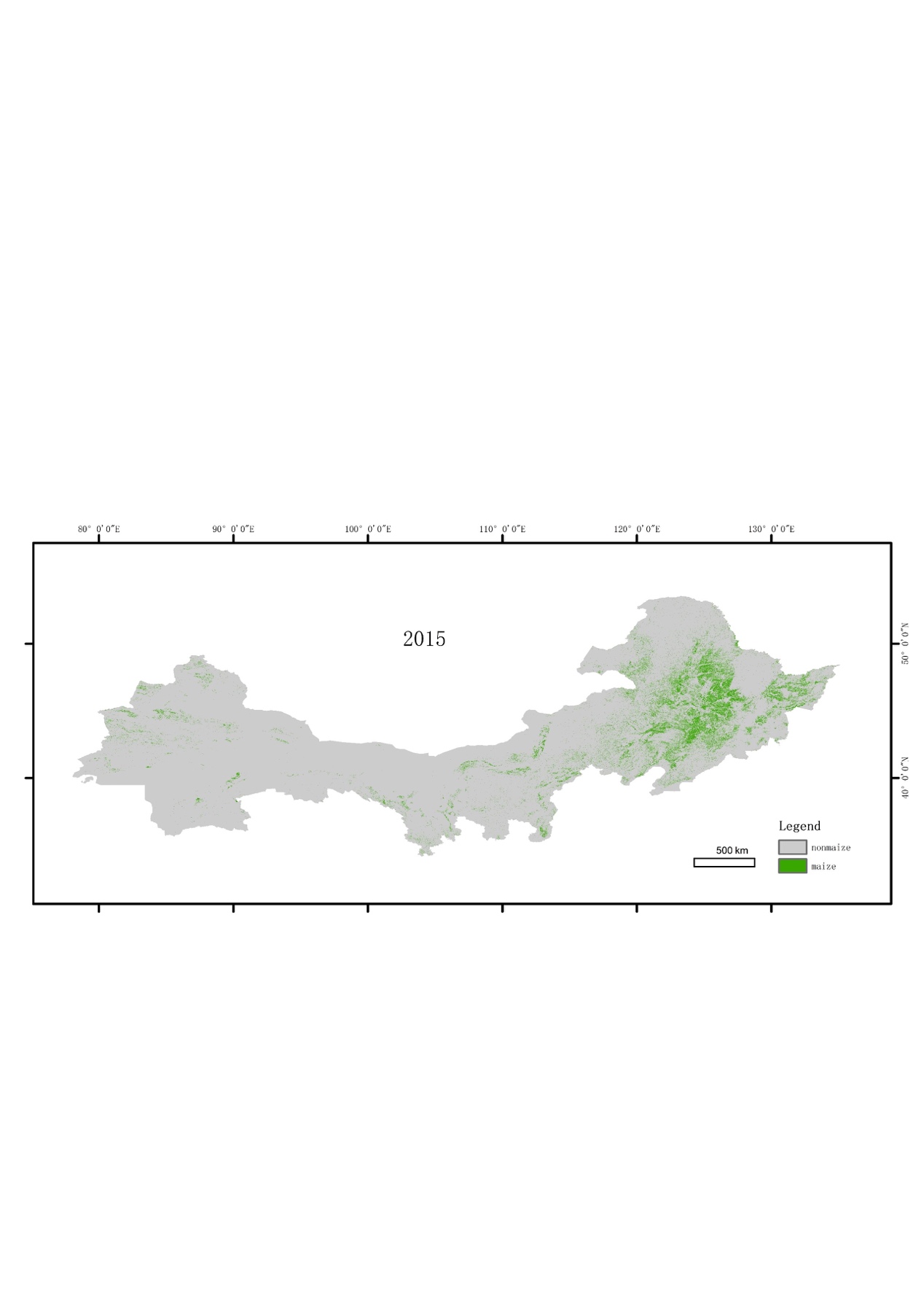


(c)


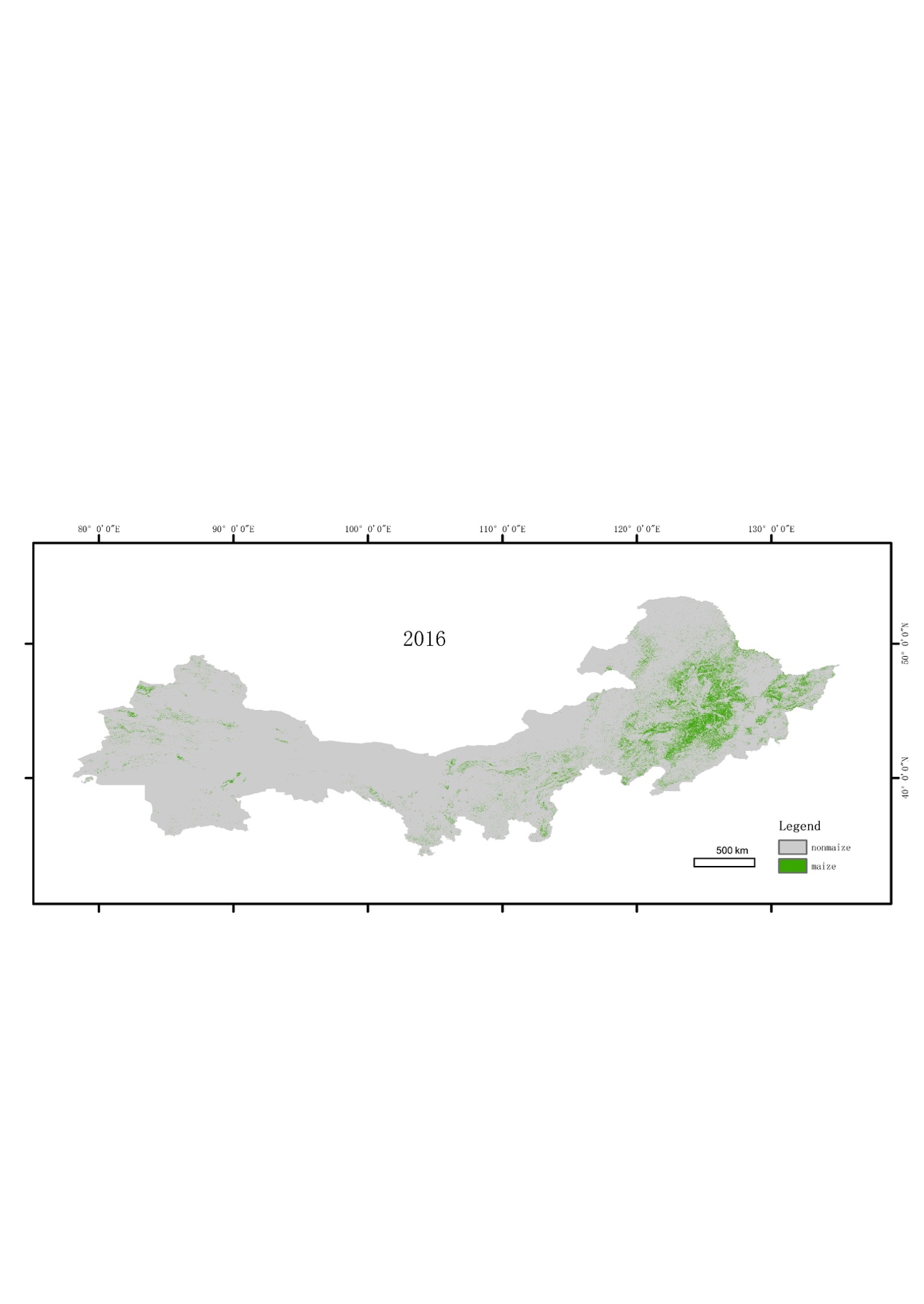


(d)


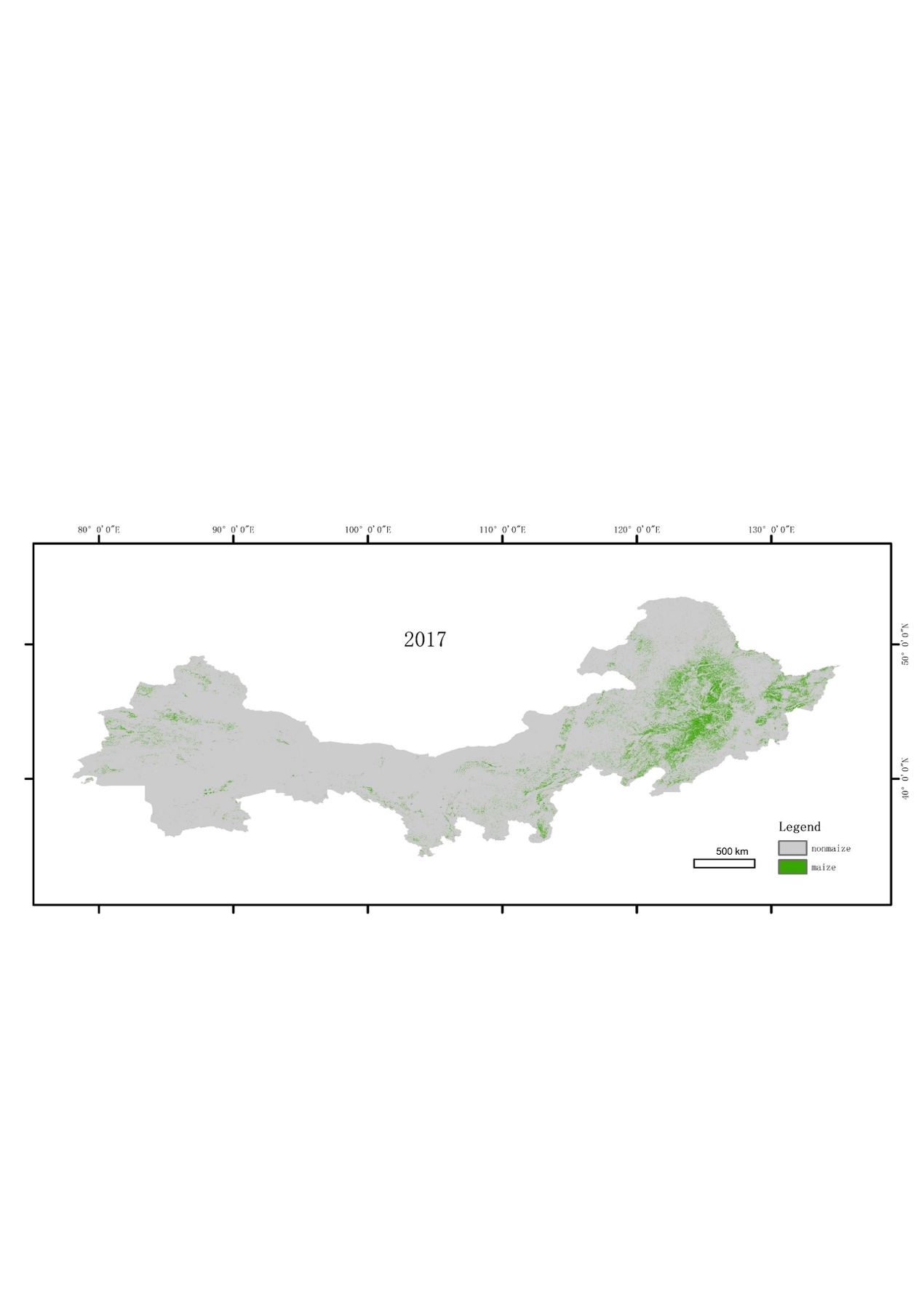


(e)


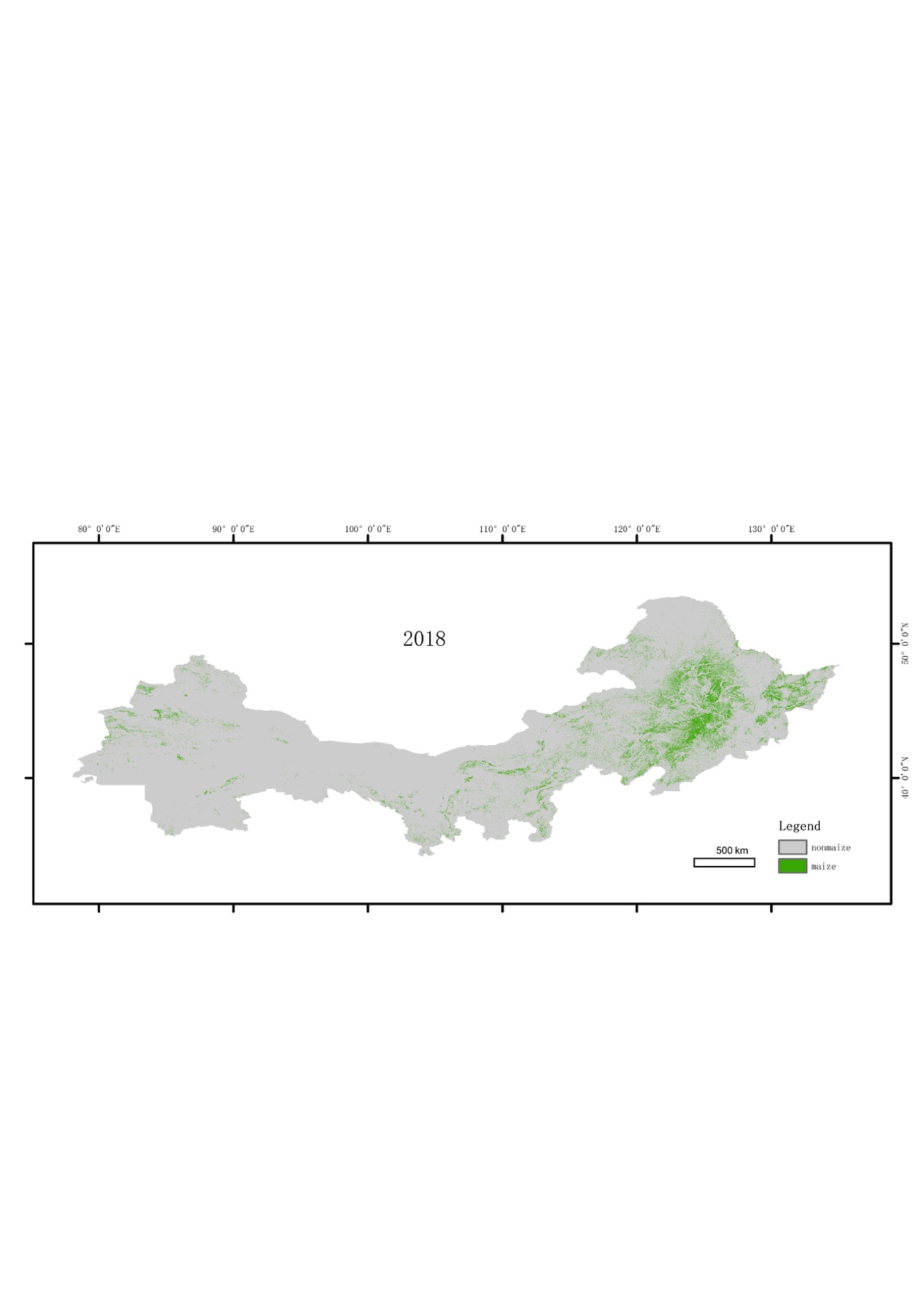


(f)


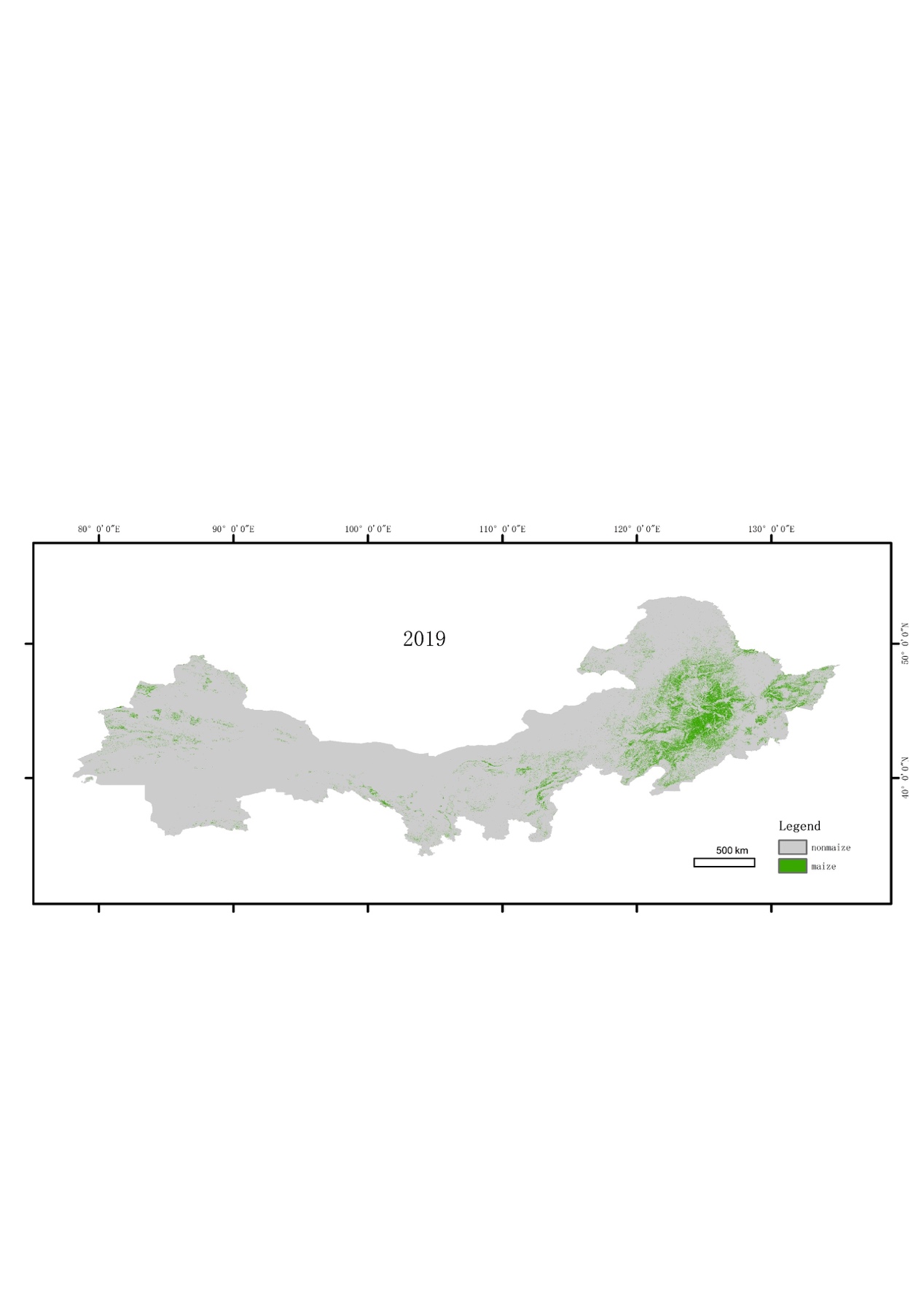


(g)


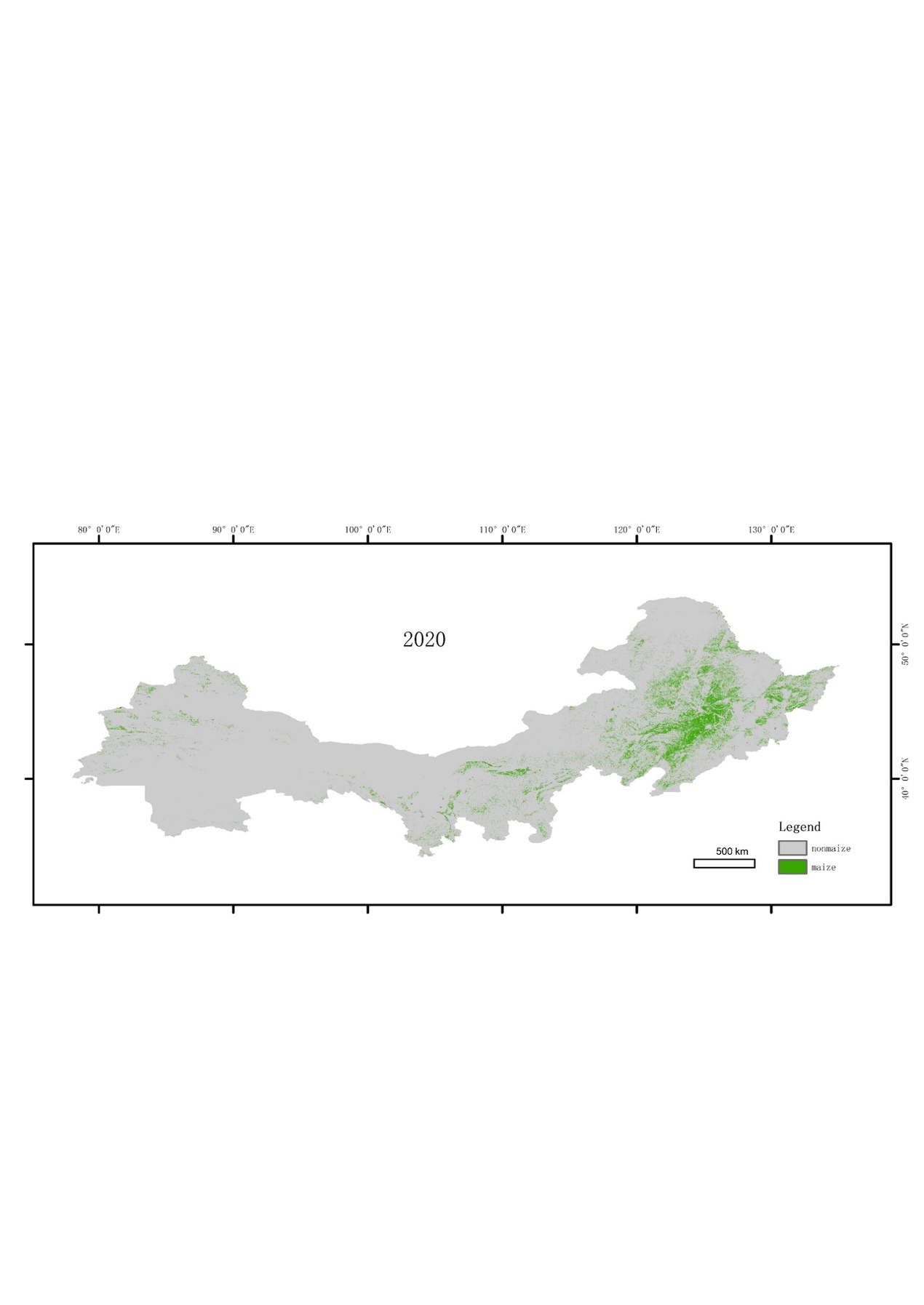


(h)


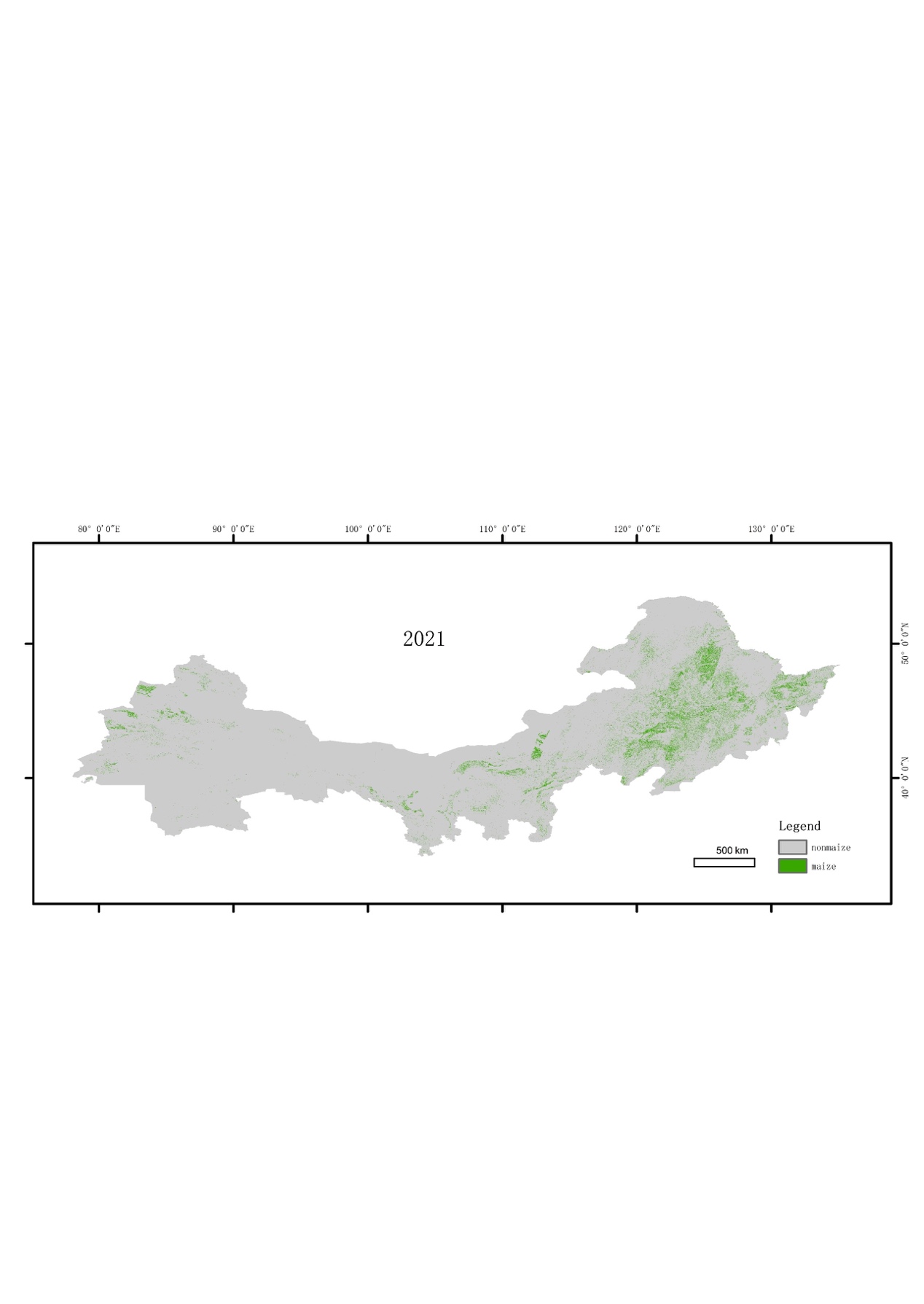


(i)

**Fig. S2** Crop maps of China’s single crop areas for 2013-2021. (a)-(i) The generated maps for each year from 2013-2021.
